# ﻿First record of *Buninotus* Maldonado Capriles and *B.palikur* Castro-Huertas, Forero & Melo from Brazil, with taxonomic notes (Hemiptera, Reduviidae, Emesinae) and an updated key to the genera of Saicini of the New World

**DOI:** 10.3897/zookeys.1213.118594

**Published:** 2024-09-25

**Authors:** Hélcio R. Gil-Santana, Jader Oliveira

**Affiliations:** 1 Laboratório de Diptera, Instituto Oswaldo Cruz, Av. Brasil, 4365, 21040-360, Rio de Janeiro, RJ, Brazil Laboratório de Diptera, Instituto Oswaldo Cruz Rio de Janeiro Brazil; 2 Universidade de São Paulo, Faculdade de Saúde Pública, Laboratório de Entomologia em Saúde Pública, São Paulo, SP, Brazil Universidade de São Paulo São Paulo Brazil; 3 Programa de Pós-Graduação em Ciência, Inovação e Tecnologia para a Amazônia, Universidade Federal do Acre, Rio Branco, AC, Brazil Universidade Federal do Acre Rio Branco Brazil

**Keywords:** Heteroptera, Neotropics, *Oncerotrachelus*, Saicinae

## Abstract

*Buninotus* Maldonado Capriles, 1981 and *Buninotuspalikur* Castro-Huertas, Forero & Melo, 2022 (Hemiptera, Reduviidae, Emesinae, Saicini) are recorded from Brazil for the first time. Taxonomic notes on *Buninotus* and its species are provided mainly based on the examination and photographs of the holotype and paratype of *Buninotusniger* Maldonado Capriles, 1981. Previous doubts on some characteristics of the genus are clarified. A hypothesis suggesting that the holotype and paratype of *B.niger* may belong to different species is presented. An updated key to the New World genera of Saicini is provided.

## ﻿Introduction

Reduviidae is one of the largest and most diverse family of predaceous Heteroptera, comprising approximately 7,000 species distributed across about 20 subfamilies worldwide (Gil-Santana et al. 2015; Schuh and Weirauch 2020; Standring et al. 2023). A new classification to the subfamily Emesinae and some closely related subfamilies was proposed by Standring et al. (2023), which resulted in Saicinae and Visayanocorinae (the latter not occuring in the New World) being considered as tribes of Emesinae. Additionally, the former emesine tribes Ploiariolini and Metapterini were treated as junior synonyms of Emesini, resulting in Emesinae sensu nov. having six tribes: Collartidini, Emesini, Leistarchini, Oncerotrachelini (as a new tribe), Saicini, and Visayanocorini.

Therefore, there are currently 10 genera of Saicini in the New World, three of which are currently monotypic (Gil-Santana et al. 2015; Castro-Huertas et al. 2023). Gil-Santana et al. (2015) provided a summary of the taxonomy of this group. Several keys to New World genera of Saicinae have been presented in the last four decades (e.g. Maldonado Capriles 1981; Blinn 1990; Melo and Coscarón 2005; Gil-Santana et al. 2006; Weirauch and Forero 2007a; Gil-Santana and Costa 2009; Gil-Santana et al. 2015, 2020). However, due to changes in the group, now considered as a tribe, including the exclusion of the genus *Oncerotrachelus* Stål, 1868 and new information on the genera *Buninotus* Maldonado Capriles, 1981 and *Caprilesia* Gil-Santana, Marques & Costa, 2006 (Castro-Huertas et al. 2023; this work), all these keys have become outdated.

Little is known about the biology and natural history of Saicinae (Gil-Santana et al. 2010), and summaries or new data on this subject, have been provided by Gil-Santana et al. (2010), as well as Gil-Santana et al. (2015, 2020), Schuh and Weirauch (2020), and Castro-Huertas et al. (2023).

In addition to documenting the first records of *Buninotus* and *B.palikur* Castro-Huertas, Forero & Melo, 2022 from Brazil, the holotype and paratype females of *B.niger* Maldonado Capriles, 1981 were directly examined and photographed to record and clarify important characteristics and address any uncertainties.

An improved and updated key to the genera of New World genera of Saicini is presented.

## ﻿Materials and methods

The female holotype and paratype of *Buninotusniger* (Figs 1–15), currently deposited in the National Museum of Natural History (**NMNH**), Smithsonian Institution, Washington, DC, USA, were directly examined and photographed (Figs 1–3, 5–15) by the second author. The photograph of Fig. 4 was taken and kindly provided by Thomas Henry. Photographs were taken using a Leica DFC450 digital camera mounted on a Leica M205 C stereomicroscope. Composite images were assembled using the Leica Application Suite v. 4.5 and the Helicon Focus v. 6.2.2 software packages.

**Figures 1–5.**
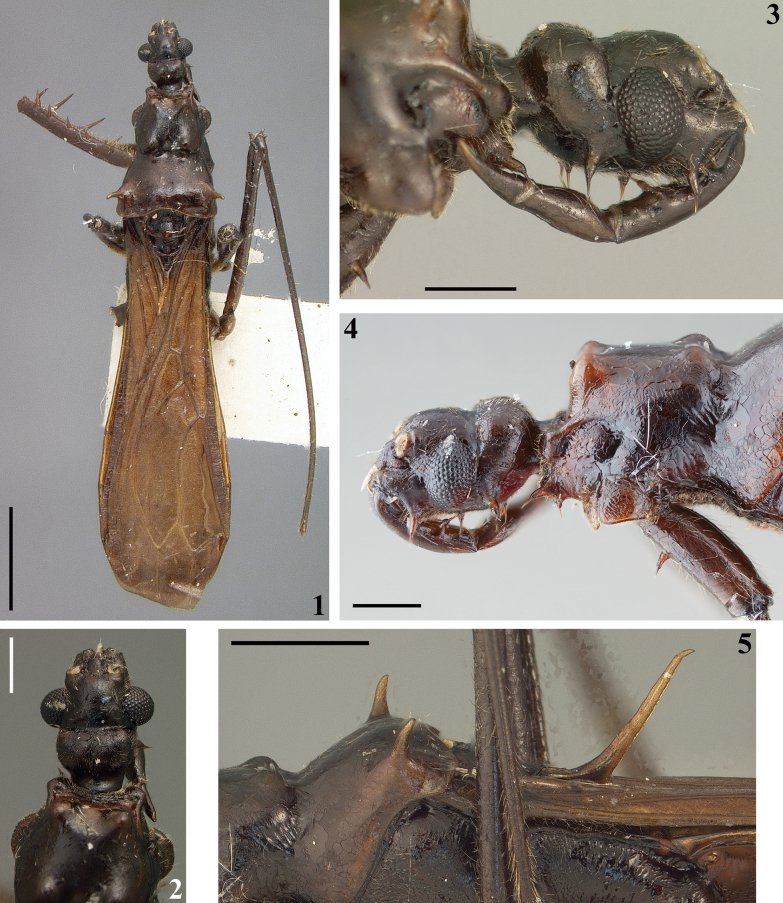
*Buninotusniger* Maldonado Capriles, 1981, female holotype deposited in NMNH**1, 2** dorsal view **3–5** lateral view **2, 3** head **4** head, anterior portion of prothorax and fore coxa **5** upper portion of hind lobe of pronotum and meso- and metathorax. Scale bars: 2.0 mm (**1**); 0.5 mm (**2–5**).

The female of *Buninotuspalikur* (Figs 16–21) from Brazil was examined and imaged by the first author. Observations were made using a stereomicroscope (Zeiss Stemi). Photographs were taken using digital cameras (Nikon D5600 with a AF-S Micro Nikkor 105 mm f/2.8G IF-ED lens and Sony DSC-W830). This specimen will be deposited in the Entomological Collection of the Museu Nacional da Universidade Federal do Rio de Janeiro, Rio de Janeiro, Brazil (**MNRJ**).

General morphological terminology primarily follows Schuh and Weirauch (2020) and Castro-Huertas et al. (2023). The latter authors introduced a new terminology, designating cuticular processes on the ventral surface of the head and labium as “spiniform setae” and “strong setae” instead of “spines,” as used by other authors. Setae without thickening were referred to as “simple setae” or simply “setae.” They argued that this revised terminology would facilitate the coding of homologous characters for future phylogenetic analyses. Therefore, for the sake of uniformity, we will adhere to this approach in the present work. The visible segments of the labium are numbered as II–IV, considering that the first segment is either lost or fused to the head capsule in Reduviidae (Weirauch 2008).

When describing label data, a slash (/) separates lines, and a double slash (//) indicates different labels. Comments or translations to English of the label data are provided in square brackets ([]).

## ﻿Results

**Subfamily Emesinae**

**Tribe Saicini**

### ﻿ Buninotus

Taxon classificationAnimaliaHemipteraReduviidae

Maldonado Capriles, 1981

F79BC4BC-532C-5A93-A8D8-6BA8B73F5BA6

#### Remarks.

In 1981, Maldonado Capriles described *Buninotus* as a monotypic genus, designating *B.niger* as its type species. He outlined the following as the main characteristics of the genus: the body is mostly black, shiny, and predominantly glabrous; a subglobose posterior lobe on the head; only the first [visible] segment of the labium is spined; scutellum with a long inclined spine; mesoscutum with a short, broad, spinelike elevation. The fore coxa, femur, and tibia are spined, with the tibia curved. The forewing exhibits four closed cells.

Some characteristics recorded in Maldonado Capriles’s (1981: 404, 406) description of the genus being a female, deserves to be mentioned, such as: the “tylus” [clypeus] as “ending in a sharp spine that slightly surpasses [the] apex of [the] jugae. ... Legs: forecoxa with a strong “s-spine near base on anterior side, 3 strong s-spines on rear of inner face; trochanter with four s-spines along inner-lower surface, femur ... with 5 s-spines along upper surface ...; lower inner surface with 5 s-spines of nearly equal size ...; tibia strongly curved on lateral aspect ..., four long s-spines on inner side, the basal the shortest ...”

Castro-Huertas et al. (2023) stated that *Buninotus* could be characterized by the first and second visible labial segments with a pair of spiniform setae and strong setae, respectively; the anterior lobe of pronotum with four protuberances [“humps”], a pair on each anterior and posterior region. The humeral angles project into long spines; scutellum has a long and inclined process. The forelegs exhibit coxae, femora and tibiae with long spiniform setae; protibiae are curved. The meso and metafemora each have a pair of apically located spiniform setae. Additionally, the forewing is characterized by three closed cells.

**Comments.** Although the venation of the wings is considered excellent for taxonomic characteristics at the generic and tribal levels in Emesinae (Wygodzinsky 1966) and has been extensively used to diagnose and/or separate supra-specific taxa, potential intra-specific variation might happen (Gil-Santana and Marques 2005) such as in the intra-specific variation in the number of cells in the forewing of *Mayemesalapinhaensis* (Wygodzinsky, 1950) (Emesini) (Gil-Santana et al. 1999).

In regard to the number of closed cells in the forewing, there is a discrepancy between Castro-Huertas et al. (2023) statement, noting three closed cells, and the description and figure by Maldonado Capriles (1981), which indicate four closed cells. Other authors, such as Gil-Santana et al. (2020), have adhered to the original description in their key for Saicinae genera. Castro-Huertas et al. (2023: 50) justified their observation by stating that they “examined an image of the holotype of *B.niger*, and it is very difficult to see the forewing vein structure without removing the forewings from the body because of the semi-hyaline to brown coloration. Using additional specimens of both *B.niger* and *B.palikur*,” they removed the forewings and found three closed cells in both species. However, it is possible that, due to the deep blackish coloration of the holotype of *B.niger*, the specimens examined by them as such may belong to a different species with a brownish general coloration (see below).

**Figures 6–8.**
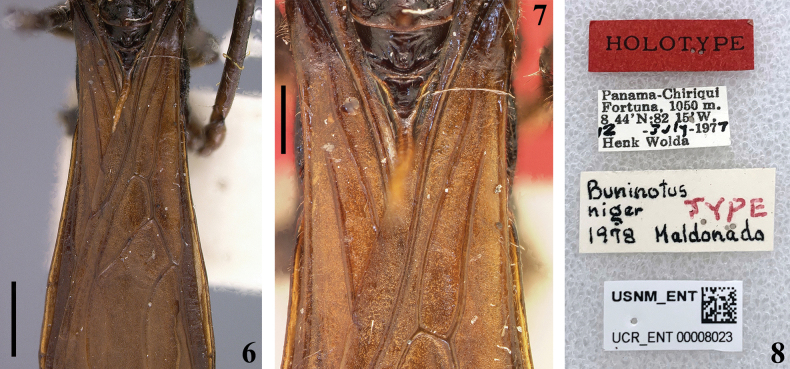
*Buninotusniger* Maldonado Capriles, 1981, female holotype deposited in NMNH**6, 7** forewings, dorsal view **6** basal two thirds **7** basal third **8** labels. Scale bars: 0.5 mm (**6, 7**).

Examination of both the holotype and paratype of *B.niger* (Figs 6, 7, 14) confirms that the veins near the base of the forewing are not united, thereby not forming a closed basal cell. Consequently, the forewing has only three closed cells in all specimens of *Buninotus* examined, supporting the observations of Castro-Huertas et al. (2023).

A striking characteristic of *Buninotus* described and illustrated by Maldonado Capriles (1981: fig. 7), but in need of confirmation, is a spine on the apex of the clypeus (“tylus”). This feature was not mentioned or questioned by Castro-Huertas et al. (2023) when discussing the characteristics of the genus. According to our observations, this spine is completely absent in both the holotype and paratype (Figs 2–4, 11). It is possible that the whitish apical portion of the labrum of the holotype of *B.niger*, which projects slightly forward (Figs 2–4), may have caused confusion for Maldonado Capriles when describing the specimen. Clearly, it is definitive that there is no apical sharp spine on the clypeus, as described by Maldonado Capriles (1981).

The presence of a pair of spiniform setae and strong setae on first and second visible labial segments, respectively, is confirmed in the holotype of *B.niger* (Fig. 3) and on a specimen of *B.palikur* from Brazil examined here (Fig. 17). In the paratype of *B.niger* the setae of the second visible labial segment is not visible (Fig. 11); they may have broken off, but it was not possible ascertain if the insertion hole of these setae are present or not because the head is covered by hyphae of mold (Figs 10–12).

**Figures 9–15.**
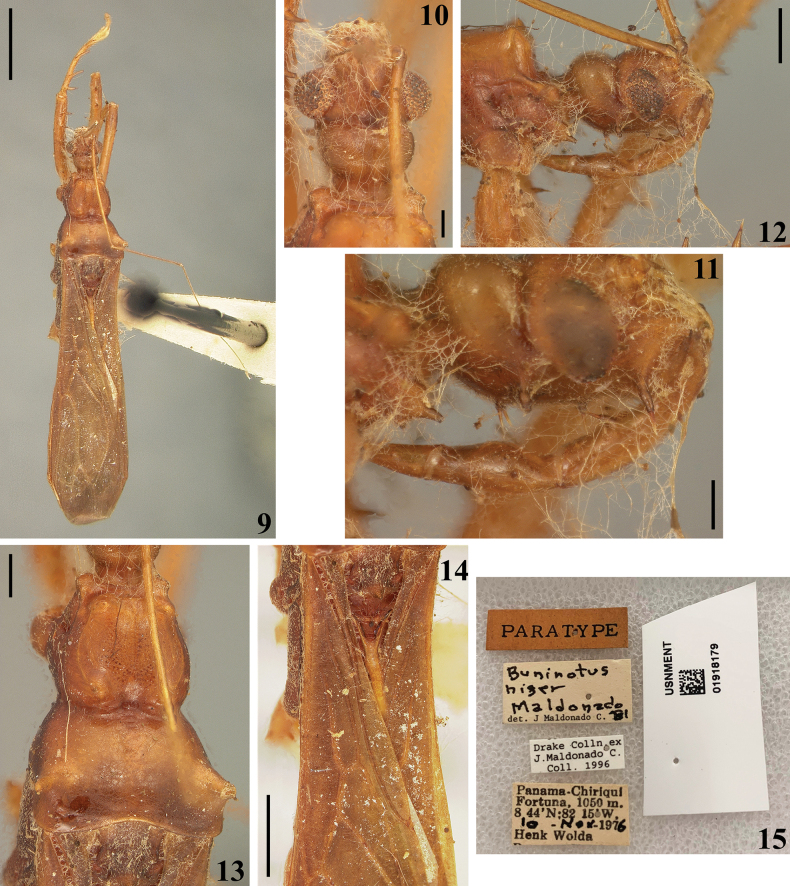
*Buninotusniger* Maldonado Capriles, 1981, female paratype deposited in NMNH**9, 10** dorsal view **10, 11** head **11, 12** lateral view **11** except upper surface **12** head and anterior portion of thorax **13, 14** dorsal view **13** pronotum **14** basal half of forewings **15** labels. Scale bars: 2.0 mm (**9**); 1.0 mm (**14**) 0.5 mm (**12**); 0.2 mm (**10, 11, 13**).

Some portions of the type specimens and the specimen from Brazil are broken or missing, mainly on the legs, but, taking into account the remaining portions and the previous descriptions (Maldonado Capriles 1981; Castro-Huertas et al. 2023) and the examination of these specimens, the number and location of spiniform/strong setae of fore legs could be added to characterization of the genus in more detail as follows: fore coxae with a dorsobasal and three ventral spiniform setae; fore trochanters with four spiniform setae, midventrally; fore femora with two rows of five spiniform setae, one row on anterodorsal portion and other on anteroventral region; fore tibiae with four anterodorsal spiniform setae, the most basal being shorter than the following ones.

#### Distribution.

Brazil (new record), French Guiana and Panama.

### ﻿ Buninotus niger

Taxon classificationAnimaliaHemipteraReduviidae

Maldonado Capriles, 1981

888C1F43-22F5-5674-9352-8C3AACE7DB78

Figs 1–5 , 6–8 , 9–15

#### Type material examined.

*Buninotusniger*. ***Female holotype***: [printed label:] Panama-Chiriqui / Fortuna, 1050 m. / 8 44'N;82 15'W, / [handwritten:] ?2 [printed:] - [handwritten:] July [printed:] -197 [handwritten:] 7 / Henk Wolda // [handwritten label:] *Buninotus* / *niger* [in red:] TYPE / 1978 Maldonado // [printed red label:] HOLOTYPE // [printed label:] [at left side:] USNM_ENT [at right side:] QR CODE / UCR_ENT 00008023; ***Female paratype***: [printed label:] Panama-Chiriqui / Fortuna, 1050 m. / 8 44'N;82 15'W, / [handwritten:] 10 [printed:] - [handwritten:] Nov [printed:] -197 [handwritten:] 6 / Henk Wolda // [handwritten:] *Buninotus* / *niger* / Maldonado / [printed:] det. J. Maldonado C. [handwritten:] 81 // [printed label:] Drake Colln ex / J. Maldonado C. / Coll. 1996 // [printed red faded label:] PARATYPE // [printed label:] USNMENT / QR CODE / 01918179 (NMNH).

Maldonado Capriles (1981) described *B.niger* based on two females from Panama (Figs 1–7, 9–14). It seems like the definition of the species coloration was primarily based on the holotype, which is deep blackish and aligns with other details recorded by Maldonado Capriles (1981) (Figs 1–7). In contrast, the paratype exhibits a general pale-brownish coloration that does not match the aforementioned description (Figs 9–14).

Castro-Huertas (2023) examined four females of *Buninotus* from Panama, also identified them as *B.niger*, and noted a dark-brownish general coloration.

#### Comments.

**Figures 16–21.**
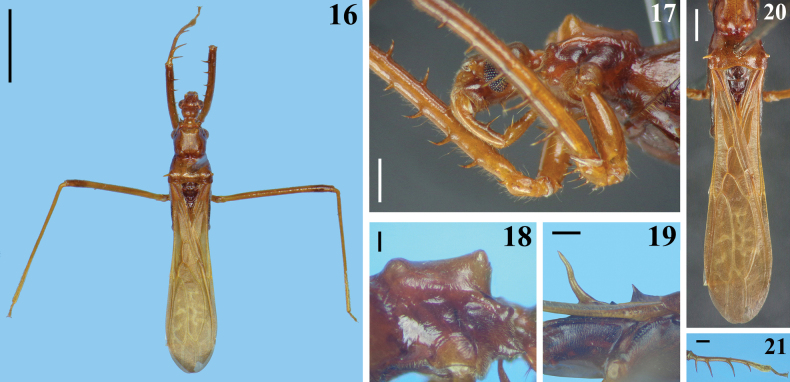
*Buninotuspalikur* Castro-Huertas, Forero & Melo, 2022, female specimen from Brazil **16** dorsal view **17** anterior portion of head, prothorax and some portions of the legs, lateroventral view **18, 19** lateral view **18** anterior portion of prothorax **19** spine of mesoscutum and scutellum **20, 21** dorsal view **20** hind lobe of pronotum and forewings **21** left fore tibia. Scale bars: 3.0 mm (**16**); 1.0 mm (**20**) 0.5 mm (**17, 19, 21**); 0.2 mm (**18**).

The holotype of *B.niger* exhibits a characteristic not observed in any other specimen of *Buninotus* so far: a deep blackish, piceous general coloration (Figs 1–6). In contrast, all other specimens of *Buninotus* display a general brownish coloration (Castro-Huertas et al. 2023; Figs 16–20), including the paratype of *B.niger* (Figs 9, 13). Additionally, the spiniform setae of the femur [only the left one was present when examined] are longer (Fig. 1) than those of the paratype (Fig. 9). Therefore, it is hypothesized here that the paratype of *B.niger* belongs to a different species than that of the holotype. In this case, the specimens identified by Castro-Huertas et al. (2023) as *B.niger* probably belong to this undescribed species. Finding more specimens, preferably including males, may help in confirming or disproving the hypothesis made here.

#### Distribution.

Panama.

### ﻿ Buninotus palikur

Taxon classificationAnimaliaHemipteraReduviidae

Castro-Huertas, Forero & Melo, 2022

0DBFCA8F-B969-5386-A9B7-F0371870AABE

Figs 16–21

#### Material examined.

Brazil, Espírito Santo: Linhares, Reserva Natural Vale [Vale Natural Reserve] (RNV), 19°09'S, 40°04'W, José Simplício dos Santos leg., 25.i.2007, 1 female (MNRJ).

#### Comments.

*Buninotuspalikur* was described based on six females from French Guiana (Castro-Huertas et al. 2023). The female examined here (Figs 16–21), although has lost its antennae and hind legs (Fig. 16), corresponds well with the description and diagnosis of *B.palikur* provided by Castro-Huertas et al. (2023). Its general length measured 10.70 mm to the tip of the membrane; the protuberances of the fore lobe are slightly triangular (Figs 16, 18), and the forewings are brownish with pale spots (Figs 16, 20). Diverging from some characteristics recorded in the specimens described by Castro-Huertas et al. (2023), the prothorax is almost uniform brownish without paler portions, the scutellar spine is curved at approximately its middle portion on lateral view (Figs 16, 18, 19), and the first spiniform setae of fore tibiae are longer (Fig. 21). However, we consider these differences as more probably intraspecific differences. If merely interindividual or geographical variations, only future examination of more specimens would allow to clarify their significance.

#### Distribution.

Brazil (new record) and French Guiana.

#### Discussion.

There is a need to collect more specimens, including males of *Buninotus*, for a better understanding of the genus and its species, possibly allowing a more comprehensive study of the systematics of Saicini in general. Finding a specimen of *B.palikur* in a natural reserve in Atlantic Forest in Brazil expands the distribution of the species and the genus *Buninotus* to a broader range of biogreographical regions.

### ﻿Key to the New World genera of Saicini, based on Weirauch and Forero (2007a, 2007b), Gil-Santana and Costa (2009), Gil-Santana et al. (2015, 2020), and Castro-Huertas et al. (2023)

**Table:** 

1	Foreleg without spiniform setae, at most with erect setae	**2**
–	Fore femur with two or three rows of spiniform setae, fore tibiae either with setae or with spiniform setae	**4**
2	Opposed surfaces of labium and head with spinelike setae or bristles; forewing with two or three cells; metapleura without a tubercle near coxal cavity	**3**
–	Opposed surfaces of labium and head without spiniform setae or bristles; forewing with four cells; metapleura with a tubercle near coxal cavity	***Saicireta* Melo & Coscarón, 2005**
3	Process on lower anterior angle of prothorax acute to subacute; second antennal segment about half as long as the first antennal segment; medial process of male pygophore bifurcate; posterior margin of seventh abdominal sternite in females vertical or subvertical	***Saica* Amyot & Serville, 1843**
–	Process on the lower anterior angle of the prothorax subconical; second antennal segment about 3/4 as long as the first antennal segment; medial process of male pygophore a single, erect barbless spine; posterior margin of seventh abdominal sternite in females sloping ventrocephalad	***Pseudosaica* Blinn, 1990**
4	Humeral angles of pronotum without processes, rounded	**5**
–	Humeral angles of pronotum with spinelike processes	**6**
5	Ventral portion of the head below (between) the eyes spineless; fore tibiae with a three or four (*T.femorata*) stronger, spiniform setae implanted on anterodorsal portion	***Tagalis* Stål, 1860**
–	Head with a ventral pair of spiniform setae below (between) the eyes; fore tibiae with a single or double longitudinal row of numerous short spiniform setae on median portion of inner surface	***Quasitagalis* Gil-Santana, Oliveira & Zampaulo, 2020**
6	Fore coxae and anterior pronotal lobe unarmed	***Bagriella* McAtee & Malloch, 1923**
–	Fore coxae spined, anterior pronotal lobe with four spines or rounded humps	**7**
7	Fore lobe of pronotum with four spines	***Paratagalis* Monte, 1943**
–	Fore lobe of pronotum with four rounded protuberances	**8**
8	Fore tibiae with a row of very short spiniform setae directed mediad; only apterous females known	***Kiskeyana* Weirauch & Forero, 2007**
–	Fore tibiae with three to six more or less large spiniform setae on anterodorsal surface, directed anteriad; all known females macropterous	**9**
9	Ventral surface of labium: first visible segment with a pair of spiniform setae, second segment with a pair of strong setae; third segment without setae. Forewings with three closed cells	***Buninotus* Maldonado, 1981**
–	Ventral surface of labium: first and second visible labial segments with a pair of spiniform setae, third segment with a pair of strong setae. Forewings with two closed cells	***Caprilesia* Gil-Santana, Marques & Costa, 2006**

Recently Castro-Huertas and Melo (2023) have recovered *Saiciretacorrentina* Melo & Coscarón, 2005 as sister species of some groups, including the clade Saicinae*sensu stricto*. However, they did not formally rule out *Saicireta* from Saicinae (Saicini), and neither did they include it in another taxonomic group. Therefore, because it has remained in Saicini, it was included in the key to this group.

## Supplementary Material

XML Treatment for Buninotus

XML Treatment for Buninotus niger

XML Treatment for Buninotus palikur

## ﻿Additional information

### Author ORCIDs

Hélcio R. Gil-Santana https://orcid.org/0000-0002-0544-348X

Jader Oliveira https://orcid.org/0000-0002-2588-1911

### Data availability

All of the data that support the findings of this study are available in the main text.
